# Modelling for Integration With Proteins, Networks and Signals

**DOI:** 10.1002/cfg.361

**Published:** 2004-02

**Authors:** Ray Paton

**Affiliations:** BioComputing and Computational Biology Group Department of Computer Science University of Liverpool Liverpool UK


**Comparative and Functional Genomics**
*Comp Funct Genom* 2004; **5**: 50–51.
Published online in Wiley InterScience (www.interscience.wiley.com). **DOI:** 10.1002/cfg.361

**Editorial**

### Modelling for integration with proteins, networks and signals

### Introduction

The articles presented in this section are based on talks given to the fourth meeting of the Modelling for Integration with Proteins, Networks and Signals (MIPNETS) Network (**http://www.csc.liv.ac.uk/~rcp/mipnets/**). This Network is funded by the UK Engineering and Physical Science Research Council as part of its Life Science Interface Initiative. The Network has drawn together biologists, computer scientists, mathematicians, medics and physiologists to explore issues concerned with transferring computational and mathematical tools and techniques to problems concerned with protein interactions, networks and signals.

**Beynon** considers future developments for proteomics toolkits. He notes that although several sophisticated tools for data reduction and analysis are available, they lack the flexibility to cope with increasingly innovative experimental strategies and new database resources that encode both qualitative and quantitative data. He outlines a specification for a flexible proteomics tool that could address many current bottlenecks and deficiencies. The need for integrative toolkits is also discussed by **Pettifer** *et al.*, who describe the UTOPIA project, which is concerned with building reusable software components that can be combined to make useful applications in the field of bioinformatics. Expertise in the fields of human computer interaction, high-performance rendering and distributed systems is being guided by bioinformaticians and end-user biologists to create a toolkit that is both architecturally sound from a computing point of view, and directly addresses end-user and application-developer requirements.

We continue with several papers that are concerned with the analysis of data. The first, by **Skilling** *et al.*, introduces and describes a fragmentation model for the interpretation of electrospray tandem mass spectrometry data. **Atreas** *et al.* report on work that uses the immune system as a metaphor for computational problem solving. They use wavelet-type discrete transforms for signal analysis on strings of finite length, and apply these transforms for edge and for hidden Markov process detection. New approaches for string matching and for measures of the diversity of chaotic strings are also discussed. **Patel and Nagl** introduce the MicroCore toolkit, which is a suite of analysis programs for microarray and proteomics data. The application implements two programs — Protein Interaction Maps (PIMs) and MicroExpress. These programs provide a simple yet powerful way of graphically relating large quantities of expression data from multiple experiments to cellular pathways and biological processes in a statistically meaningful way.

Moving from tools and techniques, we now look at ways of articulating protein function. **Amoutzias** *et al.* examine the evolution of protein interaction networks in regulatory proteins. Combining phylogenies with network analysis, they investigate the evolutionary history of interaction networks from the bHLH, NR and bZIP transcription-factor families. The bHLH and NR networks show a hub-like structure with varying gamma values. Mutation and gene duplication play an important role in adding and removing interactions. They conclude that in several of the protein families they have studied, networks have primarily arisen by the development of hetero-dimerizing transcription factors (which have a small number of interaction partners) from an ancestral gene that interacts with any of the newly emerging proteins but also homo-dimerizes. **Paton and Toh** examine certain aspects of protein functionality with relation to some key organizing ideas. This is important from a computational viewpoint in order to take account of both modelling biological systems and knowledge of these systems. They look at some of the lexical dimensions of the function and how certain constructs can be related to underlying ideas. The pervasive computational metaphor is then discussed in relation to protein multifunctionality, and the specific case of von Willebrand factor as a 'smart' multifunctional

Copyright © 2004 John Wiley & Sons, Ltd.

protein is briefly considered. Some diagrammatic techniques are then introduced to better articulate protein function.

The next two articles are concerned with linguistic dimensions to describing cellular systems. The paper by **Gheorge and Mitrana** presents an overview of computational biology and surveys some of the natural computing models using, in both cases, a formal language-based approach. Specific reference is given to L and P systems, and X-machines. The article by **Giavitto** *et al.* presents a short introduction to the use of a rewriting system, a notion developed in computer science, as a framework for modelling and simulation of various biological processes, especially at a cellular level. They provide a brief survey of the use of algebraic rewriting systems for modelling and simulating various biological processes, particularly at the cellular level.

We conclude with an article by **Vlachos** *et al.*, which describes two approaches to the individual-based modelling of bacterial ecologies and evolution using computational tools. The first approach is a fine-grained model that is based on networks of interactivity between computational objects representing genes and proteins. The second approach is a coarser-grained, agent-based model, which is designed to explore the evolvability of adaptive behavioural strategies in artificial bacteria represented by learning classifier systems. The structure and implementation of these computational models is discussed, and some results from simulation experiments are presented. Finally, the potential applications of the proposed models to the solution of real-world computational problems, and their use in improving our understanding of the mechanisms of evolution, are briefly outlined.

### Historical comment

MIPNETS grew out of a previous EPSRC network, The CytoCom Network [1], which was concerned with Emerging Computing Paradigms. CytoCom was itself a UK development from the established series of international workshops, Information Processing in Cells and Tissues (IPCAT).

### Acknowledgements

Our thanks to the EPSRC for sponsoring MIPNETS and also to the supporters of the June 2003 event: Micromass (Waters), The British Computer Society, The Consortium for Post Genome Sciences, The Biochemical Society, E-science North West and The Department of Computer Science, University of Liverpool.

### Reference

1. Paton R, Bolouri H, Holcombe M, Parish JH, Tateson R. 2003. *Computation in Cells and Tissues: Perspectives and Tools of Thought*. Series in Natural Computing. Springer: Heidelberg.

**Ray Paton**
*BioComputing and Computational Biology Group, Department of Computer Science, University of Liverpool, Liverpool, UK*

Copyright © 2004 John Wiley & Sons, Ltd.

